# Supplementary material for: Black Tea Reduces Diet-Induced Obesity in Mice via Modulation of Gut Microbiota and Gene Expression in Host Tissues
Source: Nutrients. 2022 Apr 14;14(8):1635. doi: 10.3390/nu14081635 (PMC9027533; doi:10.3390/nu14081635)
Supplement: Supplementary file 1 [file nutrients-14-01635-s001.zip › Supplementary Information.pdf]

Supplementary Information

Black tea reduces diet-induced obesity in mice via modulation of gut microbiota and gene expression in host tissues

Liu et al.

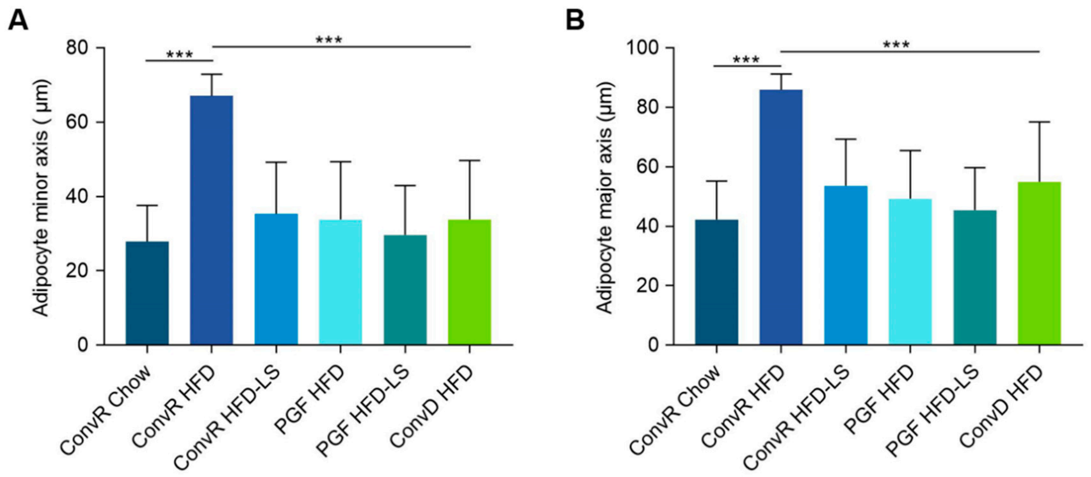

Supplementary Figure S1 The minor axis (**A**) and the major axis (**B**) of epididymal adipocyte. adipocyte size was estimated using the Image J software.

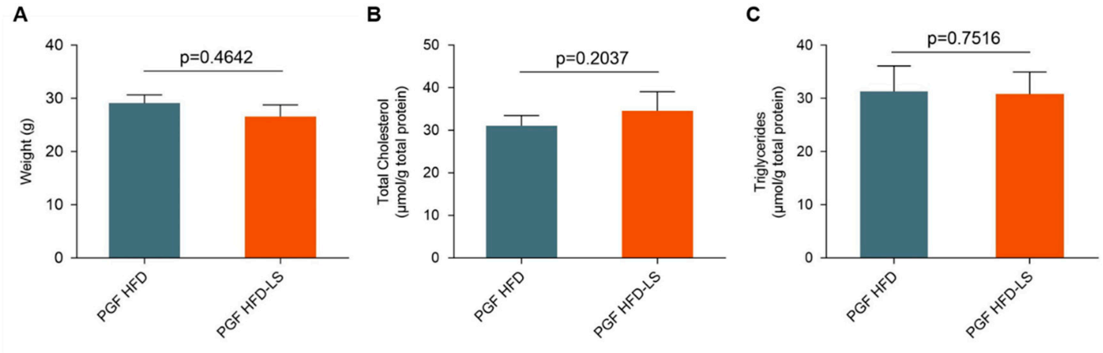

Supplementary Figure S2 Body weight and lipid lowering effects of LS alone. Weights (**A**) hepatic total cholesterol (**B**) hepatic triglycerides (**C**) of HFD-fed PGF mice treated with water or LS at the time of sacrifice. Weights data (**A**) were expressed as mean ± SD. Hepatic total cholesterol (**B**) and hepatic triglycerides (**C**) data were expressed as mean ± SEM. Differences of data in mice were analyzed using unpaired two-tailed Student's t-test. n = 6 mice per condition.

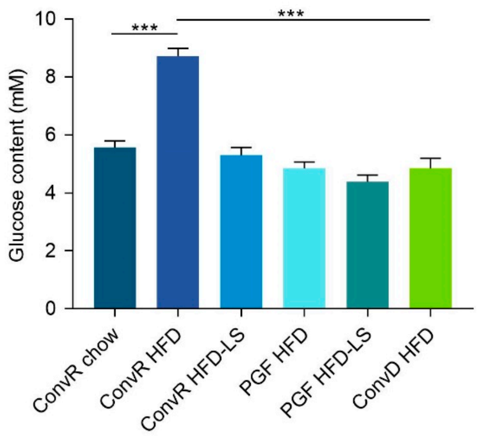

Supplementary Figure S3 Fasting glucose of Chow- and HFD-fed ConvR, PGF or ConvD mice treated with water or LS. Blood glucose of mice was determined after fasted overnight (12h). Data were expressed as mean ± SEM. Differences of data in mice were analyzed using one-way ANOVA analysis ***$p < 0.001$; $n = 6$ mice per condition.

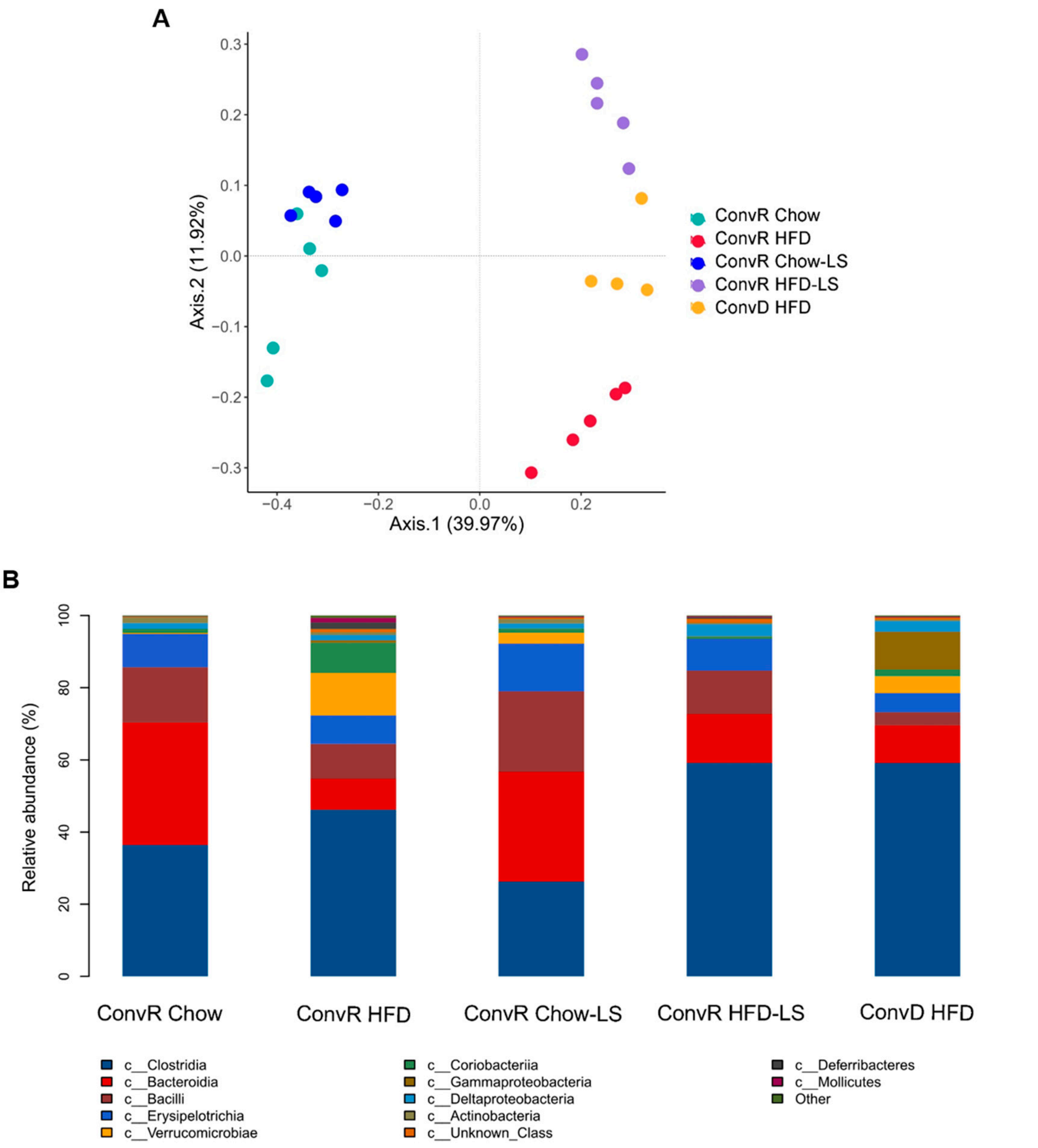

Supplementary Figure S4 Microbe communities of ConvD HFD mice were more similar to their donors. **A** Principle coordinate analysis (PCoA) plot based on the Bray-curtis distance OTU matrix of mouse colon microbiota in ConvR Chow, ConvR Chow-LS, ConvR HFD, ConvR HFD-LS and ConvD HFD groups. n = 5 mice per condition. **B** Bacterial taxonomic profiling in the class level of intestinal bacteria from different mouse groups.

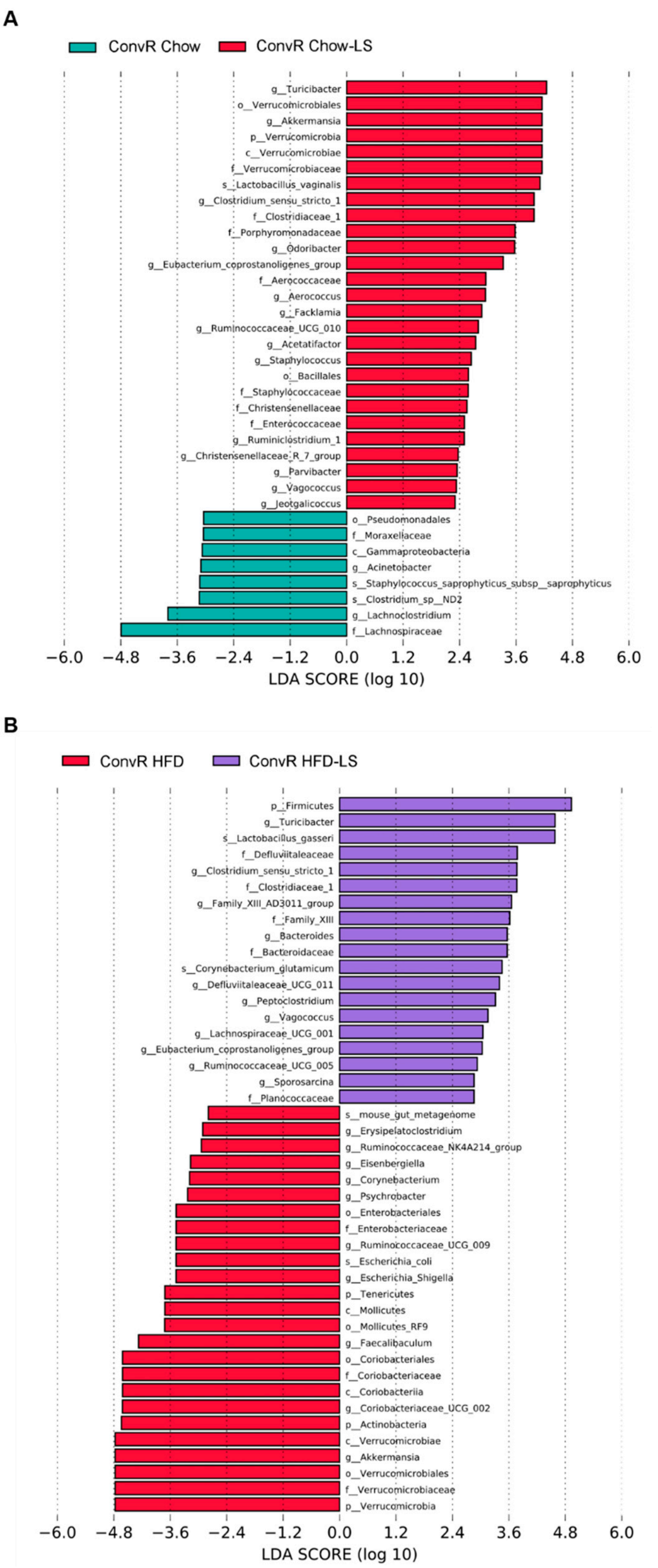

Supplementary Figure S5 LEfSe analysis on microbes in mice colon microbiota on LS consumption. LS induced differential microbes, identified by LEfSe analysis, at different phylogenetic levels in mouse colon. n = 5 mice per condition. LEfSe were conducted on ConvR Chow to ConvR Chow (**A**) and ConvR HFD to ConvR HFD-LS (**B**).

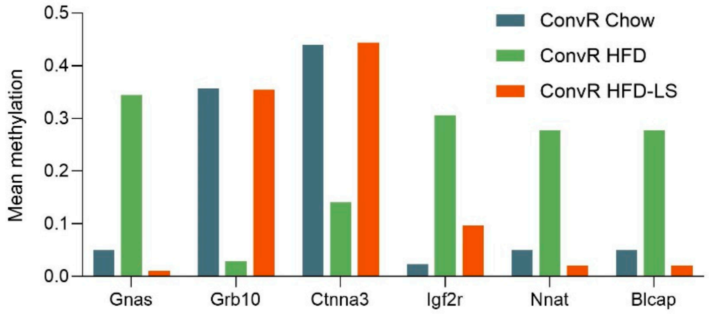

Supplementary Figure S6 DNA methylation level of imprinted genes reversed by LS treatment. Mean methylation level of imprinted genes in spermatozoa of mice. n = 11~12 mice per condition combine for one sample.

Supplementary Table S1 analysis of similarities (ANOSIM) between different groups

Group	R statistic	p-value
ConvR Chow-ConvR HFD	1	0.011
ConvR Chow-ConvR Chow-LS	0.476	0.011
ConvR Chow-ConvR HFD-LS	1	0.008
ConvR Chow-ConvD HFD	0.892	0.004
ConvR HFD-ConvR Chow-LS	1	0.01
ConvR HFD-ConvR HFD-LS	0.848	0.013
ConvR HFD-ConvD HFD	0.34	0.011
ConvR Chow-LS-ConvR HFD-LS	1	0.009
ConvR Chow-LS-ConvD HFD	0.708	0.014
ConvR HFD-LS-ConvD HFD	0.472	0.008
all	0.7502	0.001

R-value is between (-1, 1). $R>0$ represent the significant separation between groups. $R<0$ represent the intra-group difference was greater than the inter-group difference. $p<0.05$ indicate statistical significance.

Supplementary Table S2 DMGs associated with obesity and T2D in ConvR HFD vs.ConvR Chow

Obesity				T2D			
**CHR**	**start**	**end**	**Nearest gene**	**CHR**	**start**	**end**	**Nearest gene**
16	17201395	17201498	*Ube2l3*	7	19495098	19495153	Exoc3l2
16	22439666	22439718	*Etv5*	9	107908203	107908359	Mst1r
10	127571201	127571259	*Lrp1*	12	73699232	73699406	Prkch
17	27587535	27587587	*Nudt3*	18	39363303	39363387	Arhgap26
8	114550081	114550273	*Wwox*	11	95077029	95077183	Itga3
8	117343560	117343719	*Cmip*	11	57773870	57773935	Galnt10
8	124310537	124310603	*Galnt2*	16	22439666	22439718	Etv5
9	35112187	35112244	*St3gal4*	17	27587535	27587587	Nudt3
18	5591455	5591524	*Zeb1*	8	114550081	114550273	Wwox
12	7977338	7977487	*Apob*	8	117343560	117343719	Cmip
11	60199946	60200485	*Srebf1*	3	98013414	98013470	Notch2
17	35058579	35059350	*Ddah2*				
5	136567351	136567428	*Cux1*				
17	34572506	34572564	*Notch4*				
1	34291773	34291823	*Dst*				
4	133043145	133043476	*Ahdc1*				
8	120549741	120549918	*Gse1*				

Supplementary Table S3 DMGs associated with obesity and T2D in ConvR HFD-LS vs.ConvR HFD

obesity				T2D			
**CHR**	**start**	**end**	**Nearest gene**	**CHR**	**start**	**end**	**Nearest gene**
4	99194118	99194309	*Atg4c*	7	19495098	19495153	*Exoc3l2*
8	115707514	115707582	*Maf*	10	20478420	20478472	*Pde7b*
9	21735299	21735352	*Ldlr*	16	10977956	10978026	*Litaf*
5	125319233	125319414	*Scarb1*	14	25624662	25624801	*Zmiz1*
8	122893596	122893652	*Ankrd11*	8	120486526	120486593	*Gse1*
11	113709338	113709451	*Cpsf4l*	8	115707514	115707582	*Maf*
8	120486526	120486593	*Gse1*				
9	119146992	119147057	*Dlec1*				
6	28928071	28928266	*Snd1*				

Supplementary Table S4 Differentially methylated genes (DMGs) level reversed by LS treatment

Gene name	Mean methylation difference		mRNA log2(fold change)	
	ConvR HFD vs. ConvR Chow	ConvR HFD vs. ConvR HFD-LS	ConvR HFD vs. ConvR Chow	ConvR HFD vs. ConvR HFD-LS
Sap30l	0.222251	0.218578	0.079795	-0.14475
2010001A14Rik	0.222251	0.218578	-0.68558	-0.39901
Rpa1	0.535118	0.503795	-0.0275	0.510131
Gid8	0.515476	0.463102	-0.09369	0.029782
Slc35d1	0.252302	0.185363	-0.17331	-0.45417
Gm2287	0.354407	0.392552	-	-
Cenpc1	0.354407	0.392552	0.663175	0.586715
Gm43514	0.252354	0.25391	0	-1.34467
Herc2	0.16444	0.135934	-0.37196	0.225517
Ttbk1	0.419729	0.375876	1.281797	2.066431
Pdyn	-0.37455	-0.46604	-	-
Ahi1	0.29494	0.270512	0.465466	0.960975
Smyd4	0.535118	0.503795	-0.04212	-0.1701
Cep97	0.31118	0.182524	0.365471	0.51131
Utp14b	0.677732	0.509795	-0.30288	0.054473
Dido1	0.515476	0.463102	-0.40907	0.183905
Stmn1	0.499569	0.43895	0.160779	-1.48941
Arhgef10l	-0.08353	-0.27815	-0.09557	0.163344
Tacc2	0.121836	0.291786	0.623844	0.751146
Sema3f	0.251004	0.233754	-0.08934	0.018601
Hhipl1	-0.22602	-0.42439	-0.18664	-0.68216
Adamts4	-0.15209	-0.23046	1.361904	1.431656
Nelfb	0.298615	0.293398	0.157815	0.239799
Abraxas1	0.252354	0.25391	0.322562	-0.53861
2210016L21Rik	0.423121	0.42929	-0.26307	-0.99349
Exoc3l2	-0.23882	-0.19841	-0.28517	-0.1338
Pdp2	0.43306	0.409519	-0.47181	0.664233
Xpo7	0.325687	0.294298	-0.21222	0.525181
Dcbld2	-0.27484	-0.26177	-0.02702	-0.19322
Acsl3	0.677732	0.509795	0.473539	-0.00998
Ndufs2	-0.15209	-0.23046	-0.04511	0.290211
Prkcz	0.65612	0.045706	0.04402	0.217845
Fgfr2	0.121836	0.291786	-0.13994	0.006707
Arhgef7	0.195537	0.210212	0.234608	-0.1347
Prtg	0.497865	0.47779	-1.40849	-3.18037

Supplementary Table S5 Imprinted genes DNA methylation changed by HFD

CHR	start	end	length	Num_C	meanMethy ConvR	meanMethy ConvR HFD	diff_Methy	Imprinted genes
2	174299492	174299551	60	8	0.04961008	0.344423	-0.295	Gnas
7	62377549	62377632	84	6	0.46505547	0.134832	0.33	Magel2
2	157560373	157561445	1073	13	0.04970104	0.277703	-0.228	Nnat
7	6977080	6977185	106	10	0.55982551	0.235473	0.324	Zfp264
11	12037034	12037086	53	14	0.35684092	0.029114	0.328	Grb10
11	22972625	22972702	78	7	0.35656093	0.031605	0.325	Zrsr1
10	64320443	64320529	87	6	0.4398431	0.141133	0.299	Ctnna3
8	80847050	80847212	163	7	0.71313603	0.889421	-0.176	Gab1
2	157560373	157561445	1073	13	0.04970104	0.277703	-0.228	Blcap
17	12769316	12769751	436	15	0.02310583	0.306183	-0.283	Igf2r
4	154140226	154140284	59	7	0.02007366	0.379441	-0.359	Trp73
7	6937064	6937174	111	6	0.42678429	0.891498	-0.465	Usp29
3	108145935	108145995	61	18	0.01852223	0.33289	-0.314	Gnai3
7	6977080	6977185	106	10	0.55982551	0.235473	0.324	Zim3
2	162942649	162942734	86	7	0.19318458	0.201889	-0.009	L3mbtl1
